# NF‐κB/NKILA signaling modulates the anti‐cancerous effects of EZH2 inhibition

**DOI:** 10.1111/jcmm.14500

**Published:** 2019-07-07

**Authors:** Suzann Duan, Westin K. Chan, Andrew Oman, Dominic P. Basile, Cristina M. Alvira, Iain L.O. Buxton, Cristiana Iosef

**Affiliations:** ^1^ University of Nevada Reno, School of Medicine Reno Nevada; ^2^ Stanford University School of Medicine Stanford California

**Keywords:** breast cancer, EZH2, NFkB, NKILA, regulatory crosstalk

## Abstract

A wealth of evidence supports the broad therapeutic potential of NF‐κB and EZH2 inhibitors as adjuvants for breast cancer treatment. We contribute to this knowledge by elucidating, for the first time, unique regulatory crosstalk between EZH2, NF‐κB and the NF‐κB interacting long non‐coding RNA (NKILA). We define a novel signaling loop encompassing canonical and non‐canonical actions of EZH2 on the regulation of NF‐κB/NKILA homeostasis, with relevance to breast cancer treatment. We applied a respective silencing approach in non‐transformed breast epithelial cells, triple negative MDA‐MB‐231 cells and hormone responsive MCF‐7 cells, and measured changes in EZH2/NF‐κB/NKILA levels to confirm their interdependence. We demonstrate cell line‐specific fluctuations in these factors that functionally contribute to epithelial‐to‐mesenchymal transition (EMT) remodelling and cell fate response. EZH2 inhibition attenuates MDA‐MB‐231 cell motility and CDK4‐mediated MCF‐7 cell cycle regulation, while inducing global H3K27 methylation and an EMT phenotype in non‐transformed cells. Notably, these events are mediated by a cell‐context dependent gain or loss of NKILA and NF‐κB. Depletion of NF‐κB in non‐transformed cells enhances their sensitivity to growth factor signaling and suggests a role for the host microenvironment milieu in regulating EZH2/NF‐κB/NKILA homeostasis. Taken together, this knowledge critically informs the delivery and assessment of EZH2 inhibitors in breast cancer.

## 1 INTRODUCTION

In 2018, the American Cancer Society estimates that 266,120 new cases of invasive breast cancer will be diagnosed in women in the United States.^1^ Basal‐like and triple negative tumours represent approximately 20 percent of all breast cancers and are characterized by higher rates of distant recurrence and patient mortality compared to their hormone‐responsive counterparts.^2^ The failure of existing therapeutics to target these aggressive subtypes confirms the need for a deeper understanding of the molecular mechanisms that govern therapeutic response in hormone‐resistant breast cancer.

Constitutive activation of the NF‐κB transcription factor is associated with disease progression, distant reoccurrence and reduced survival outcome in breast cancer.^3^ Basal‐like and triple negative breast cancers demonstrate elevated nuclear NF‐κB expression and activity compared to luminal subtypes with or without HER2/ErbB2 amplification.^4^, ^5^, ^6^ It is widely accepted that the IKK‐IκB axis regulates canonical NF‐κB activation. In quiescent cells, NF‐κB transcription factors are sequestered in the cytoplasm through physical association with inhibitors of NF‐κB (IκB) proteins. Extracellular stimuli, including pro‐inflammatory cytokines, induce phosphorylation of IκB kinases (IKK), which then phosphorylate IκB proteins and trigger their dissociation via ubiquitination‐mediated degradation. Degradation of IκB releases NF‐κB transcription factors and promotes their nuclear translocation to initiate various transcription programs, including those that stimulate inflammation, proliferation, epithelial‐to‐mesenchymal transition (EMT), therapeutic resistance and self‐renewal.^6^, ^7^, ^8^, ^9^, ^10^, ^11^, ^12^, ^13^ Numerous regulators of the NF‐κB system have been identified and here we focus on two signaling factors with potential functional interaction: the NF‐κB interacting long non‐coding RNA (NKILA) and enhancer of zeste homolog 2 (EZH2).

Nuclear factor‐kappa B activity is augmented under the action of EZH2, a histone methyltransferase and transcriptional repressor displaying elevated and non‐canonical transactivation activity in several cancers.^14^, ^15^, ^16^, ^17^ In the canonical pathway, EZH2 acts as the catalytic subunit of the polycomb repressive complex 2 (PRC2) and prevents gene expression by methylating histone H3 on lysine 27. In breast cancer, EZH2 acts independently of PRC2 and exerts dual functions in the presence or absence of oestrogen receptor (ER). In ER^+^ cells, EZH2 physically associates with ERα and β‐catenin to activate Wnt signaling and cell cycle progression by increasing cyclin D4 and c‐Myc expression.^18^ In the absence of ER, EZH2 interacts with the NF‐κB system components RelA and RelB to potentiate NF‐κB transcriptional activity, leading to inflammation, self‐renewal and adoption of a tumour initiating cell (TIC) phenotype.^19^, ^20^, ^21^ Moreover, EZH2 over‐expression is associated with high‐grade tumours enriched in TICs, and it has been suggested that EZH2 expression favours the transition of dormant progenitors or differentiated cells into an aggressive stem cell‐like phenotype.^22^, ^23^, ^24^ Preclinical studies of EZH2 inhibitors show promise in improving cancer status, and drugs such as tazemetostat (Epizyme) are currently being explored in Phase I and II clinical trials.

The anti‐tumoural role of NKILA, a cytoplasmic NF‐κB interacting long non‐coding RNA, has been recently demonstrated in multiple cancers, including those of the breast.^25^, ^26^, ^27^ Here, we elucidate the NF‐κB/NKILA pathway in relation to EZH2, an immediate regulator of NF‐κB activity in cancer cells. Upon challenge by pro‐inflammatory cytokines, NKILA is transcriptionally activated by NF‐κB signaling and suppresses further NF‐κB activity by physically masking IKK‐mediated IκB phosphorylation. The importance of this negative feedback loop was demonstrated in breast epithelial cells, where NKILA was shown to prevent constitutive activation of the NF‐κB pathway under pro‐inflammatory conditions. Consistent with its anti‐tumoural function, low NKILA expression is associated with breast cancer metastasis and poor patient prognosis.^25^ While EZH2 is known to promiscuously bind lncRNAs through its cooperation in the PRC2 complex, the existence of direct interaction between EZH2 and NKILA remains to be elucidated.

We hypothesized that therapeutic restriction of EZH2 alters the reciprocity of NF‐κB/NKILA signaling with potential detriment to normal development patterns observed during homeostasis. We aimed to characterize and establish the EZH2/NF‐κB/NKILA signaling axis as a novel regulator of metastasis and disease progression, with added relevance in hormone resistant breast cancers. This study informs current clinical trials of EZH2 inhibitors, including tazemetostat, as adjuvant therapeutics in the treatment of solid tumours. We report that EZH2 restriction interferes with the natural reciprocal action of NF‐κB/NKILA by altering their homeostatic levels and potentially eliciting off‐target methylation in noncancerous epithelium.

## 2 MATERIALS AND METHODS

### 2.1 Cell culture and treatments

hTERT‐HME1 cells were grown in Cascade Biologics Medium 171 (Gibco). MDA‐MB‐231 cells were grown in Leibovitz's (L‐15) + GlutaMAX Medium (Gibco) supplemented with 10% foetal bovine serum (FBS). MCF‐7 cells were grown in Minimum Essential Medium (MEM) Alpha + GlutaMAX (Gibco), supplemented with Mammary Epithelial Growth Supplement (MEGS) (Gibco, S0155), 10% FBS and 0.01mg/ml human recombinant insulin. All media were passed through vacuum filtration and were protected from contamination by adding 1X Anti‐Anti (antibiotic‐antimycotic) (Gibco). Cells were grown at 37°C at 5% CO2 in T‐75 and T‐150 vent flasks. Media was changed routinely, and cells were passaged using 0.25% Trypsin‐EDTA (1X) (Gibco). To respectively inhibit EZH2/1 or EZH2 methyltransferase function, 5μM UNC1999 (Cell Signaling Technology) or 5 μmol/L tazemetostat (Selleck Chemicals) were prepared in cell line‐specific media and administered to 80%‐90% confluent flasks for 48 hours. BAY11‐7082 inhibitor compound prepared in respective cell media (5 μmol/L) was administered for 24 hours to diminish NF‐κB activity. NKILA was inhibited by transfection of microRNA 103 using Lipofectamine RNAiMAX Reagent, per manufacturer protocol.

### 2.2 Transwell migration and invasion assays

Cell monolayers were dissociated with Trypsin, re‐suspended in respective media containing 2% FBS with and without UNC1999 added (1 µmol/L), and counted using the BioRad TC‐20 automated cell counter. Cell‐suspension was diluted to a working concentration of 5 × 10^5^ cells/mL, from which a volume of 100 µL was seeded into top chambers of transwells (6.5 mm, 8 µm pore, Corning) for a final concentration of 5 × 10^4^ cells/well. Cells were allowed to settle for 1‐hour at 37°C before top chambers were supplemented with an additional 200 µL of 2% FBS‐containing media and bottom chambers were filled with 1ml of 10% FBS‐containing media. Inserts were gently lifted up and re‐submerged in lower chamber to ensure the removal of air bubbles. Plates were incubated at 37°C for 24 hours before inserts were removed and submerged in 100% methanol for 10 minutes. Inserts were then submerged in 0.5% crystal violet for 40 minutes to stain cells. Inserts were washed to remove excess dye and non‐migratory cells were swabbed away from the topside of the membrane. Membranes were cut away and mounted onto slides with immersion oil. For invasion assay, transwells were coated with 100 µL of 1 mg/mL growth factor‐reduced Matrigel^®^ diluted in ice‐cold DMEM and incubated at 37°C for 2 hours prior to seeding cells. Plates were incubated for 72 hours at 37°C and analysed as previously described. Slides were imaged using the BZ‐X700 automated fluorescence microscope (Keyence). Entire membranes were imaged at 4X and images were stitched to create a composite image for each well. Area of migration for each composite image was quantified using the hybrid cell count function on the BZ‐X700 analyzer software. Statistical analysis was performed using Graphpad Prism 7 (n = 3 per treatment). Unpaired two‐tailed *t* tests were performed to compare differences in control and UNC1999 treated cells.

### 2.3 BrdU incorporation assay

HMEC, MDA‐MB‐231 and MCF‐7 cells were seeded onto 96‐well plates (1 × 10^4^ cells/well) in respective media supplemented with 10% FBS. Cells were allowed to adhere for 3 hours at 37°C before switching to media supplemented with 2% FBS. Cells were incubated overnight in low serum (LS) conditions prior to initiating treatment. Cells were treated with respective media supplemented with 10% or 2% FBS and containing BAY11‐7082 (2.5 µmol/L), UNC1999 (1 µmol/L), Tazemetostat (5 µmol/L) or non‐treated control (0.1% DMSO). Prior to treatment, 5‐bromo‐2'‐deoxyuridine (BrdU) was added to media at a final 1X concentration. BrdU incorporation was assayed after 24 hours according to manufacturer's instructions (BrdU Cell Proliferation Kit, Cell Signaling Technology). Absorbance was measured at 450 nm using the Hidex Chameleon plate reader. Treatments were performed in quadruplicate (n = 4) and tested for statistical significance using one‐way ANOVA on Graphpad Prism 7.

### 2.4 Cell count assay

HMEC, MCF‐7 and MDA‐MB‐231 cells were seeded in triplicate (n = 3) onto 6‐well plates at a density of 0.3 × 10^6^ cells per well in complete growth medium containing 10% FBS (CGM). Cells were allowed to adhere overnight and serum starved in media containing 2% FBS (LS) for eight hours. Treatment was initiated by switching cells to CGM, LS, or LS supplemented with 100 ng/mL of human recombinant IGF‐1 (Lifeline Technologies). Following 24 hours of treatment, cells were trypsinized, quenched and counted using the TC20 automated cell counter (BioRad). Cell counts were analysed for significance by two‐way ANOVA using GraphPad Prism 7.

### 2.5 Cell lysate preparation and western blotting

Cell lysates were prepared using Cell Lysis Buffer (Cell Signaling Technology), supplemented with Phosphatase Inhibitor Cocktail 3 (Sigma) and Protease Inhibitor Cocktail (Sigma). Lysis buffer (1 mL) was added to a confluent T‐75 flask and cells were scraped off and collected for further lysing by vortexing and freeze/thawing. Protein concentrations were measured using Pierce BCA Protein Assay Kit (Thermofisher Scientific). Cell lysates (20‐30 μg) were run on NuPAGE 4%‐12% Bis‐Tris Gels (Invitrogen). Samples were run at 100 V for 1.5‐2 hours using NuPAGE MES SDS Running Buffer (Novex) and XCell SureLock Electrophoresis Cell (Novex). Gels were transferred onto Invitrolon PVDF membranes (ThermoFisher Scientific) using NuPAGE Transfer Buffer (Novex) and the XCell II Blot Module (Novex) set to 30 V for approximately 1 hour. Membranes were blocked using SEA BLOCK Blocking Buffer (ThermoFisher Scientific) for 45 minutes to 2 hours. Membranes were incubated with primary antibodies at 4°C overnight. Membranes were washed three consecutive times with PBS‐0.05% Tween‐20. Membranes were incubated in anti‐mouse or anti‐rabbit secondary antibody for approximately 45 minutes, and the washes were repeated. Membranes were then incubated in Novex ECL HRP Chemiluminescent Substrate Reagent Kit (Invitrogen) for approximately 5 minutes and imaged using GelDoc. The following primary antibodies from Cell Signaling Technology were used: EZH2 (1:1000), NF‐κB (p65) (1:1000), Vimentin (1:250), E‐Cadherin (1:500), β‐actin (1:1000), Cdk4 (1:1000), Cyclin D1 (1:1000), pAkt (1:1000). H3K27 (1:1000) and N‐Cadherin (1:1000) primary antibodies were purchased from Abcam while EZH1 (1:1000) antibody was purchased from Novus Biologicals.

### 2.6 Quantitative analysis of NKILA transcript

Total RNA was extracted from fresh cell cultures using PureLink RNA Mini Kit (Invitrogen). NKILA cDNA template was prepared using iScript Reverse Transcription Supermix (Biorad) following manufacturer instructions. PCR amplification of the NKILA fragment was performed in two ways: semi‐quantitative RT‐PCR measured by densitometry and quantitative RT‐qPCR using Taqman. RT‐PCR was performed by using Platinum Taq polymerase (Invitrogen) and the following primers, previously reported by Liu et al^25^: NKILA (sense: 5'‐AAC CAA ACC TAC CCA CAA CG, antisense: 5'‐ACC ACT AAG TCA ATC CCA GGT G‐3'). The DNA product was amplified using a Veriti thermal cycler (Applied Biosystems) and separated by electrophoresis in Novex 20% TBE gels (Invitrogen) stained with ethidium bromide. Quantitative analysis was performed using ImageJ software. Results were confirmed using Taqman RT‐qPCR. NKILA and 18S ribosomal subunit universal control primer mixes, in addition to Taqman master mix, were purchased from Applied Biosystems. Assays were performed using the ABI 7900HT system and transcript amplification results were generated using SDS 2.4 software. NKILA FAM reporter (ID:Hs04937740_s1) was normalized to 18S control (ID:Hs99999901_s1) across technical triplicates. Taqman assays were repeated a minimum of 2‐3 times for each cell line per treatment condition. Statistical significance was determined by one‐way ANOVA or Student's *t* test as appropriate using Graphpad Prism 7.

### 2.7 Receptor tyrosine kinase signaling antibody array kit

A PathScan Antibody Array Kit (Cell Signaling Technology) was used to detect receptor tyrosine kinase (RTK) activation and signaling. The array employs an antibody capture mechanism for activated RTK molecules and downstream partners. The array was placed on glass slides and the immuno‐dot‐blot procedure was performed according to manufacture protocol. A volume of 150 μL (1 μg/μL) of cell lysate was incubated overnight at 4°C with each of the arrays. After three consecutive washes, a volume of 150 μL of 1X biotinylated detection antibody cocktail was added to each well and arrays were incubated on an orbital shaker for 1 hour. To reveal the immuno‐complexes, 150 μL 1X HRP‐linked streptavidin was added to each well and incubated at room temperature for 30 minutes. After three subsequent washes, the chemiluminescence agent, LumiGLO, was added onto the arrays and images were captured at varying exposure times using a Biorad imager. Each biomarker was represented in the array by a pair of dot‐blots. Spot density analysis was performed using ImageJ software and the average dot density was plotted (n = 2).

## 3 RESULTS

### 3.1 Homeostatic expression of EZH2 and NF‐κB is inversely associated with NKILA transcription and correlates with metastatic potential

**Figure 1:**
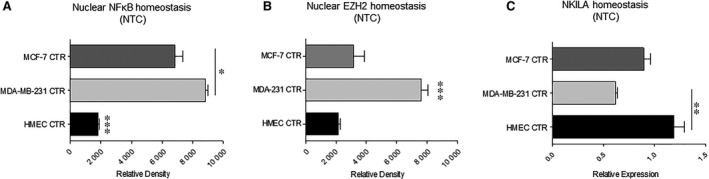
Homeostatic levels of nuclear NF‐κB, EZH2 and NKILA. Non‐treated (NTC) HMEC, MDA‐MB‐231 and MCF‐7 cells were evaluated for baseline expression of nuclear NF‐κB, EZH2 and NKILA. (A) Relative expression of NF‐κB and (B) EZH2 in nuclear lysates was measured by western blot using β‐actin as a loading control, followed by densitometry analysis of bands (n = 3). (C) Relative NKILA transcript levels were measured by quantitative real time polymerase chain reaction using 18S rRNA as an endogenous control (n = 3). *P < 0.05, **P < 0.01, ***P < 0.001 by one‐way ANOVA

We evaluated homeostatic levels of nuclear NF‐κB, NKILA and EZH2 in non‐treated (NTC) hormone receptor positive (MCF‐7), hormone receptor negative (MDA‐MB‐231) and non‐tumourigenic human mammary epithelial cells (HMEC). Nuclear NF‐κB expression was significantly elevated in both breast cancer cell lines compared to HMECs, and the highest expression was observed in MDA‐MB‐231 cells (Figure 1A). Nuclear NF‐κB levels correlated with homeostatic EZH2 expression and was inversely associated with NKILA transcript levels in all three cell lines (Figure 1B and 1). NKILA transcripts levels were lowest in MDA‐MB‐231 cells and highest in HMECs. Interestingly, levels of NKILA were lower in MDA‐MB‐231 cells compared to MCF‐7 cells, further supporting its inverse expression with nuclear NF‐κB and EZH2 in these cell lines.

### 3.2 Individual restriction of EZH2 and NKILA induce cell‐specific fluctuations in NF‐κB/NKILA/EZH2 expression levels

**Figure 2:**
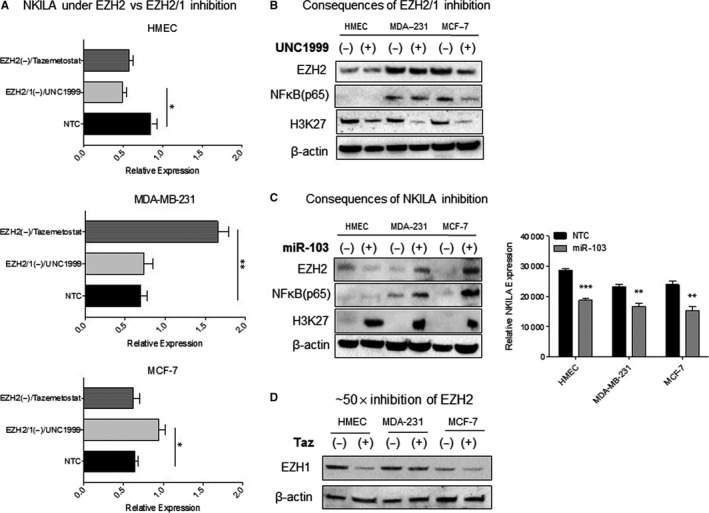
Reciprocal effects of EZH2, EZH2/1 and NKILA inhibition. (A) Relative NKILA transcript levels were evaluated by RT‐qPCR following EZH2‐specific or nonspecific EZH2/1 inhibition using tazemetostat and UNC1999, respectively (n = 3). (B) Western blots of nuclear cell lysates following EZH2/1 inhibition. (C) Western blots of nuclear cell lysates following partial silencing of NKILA under miR‐103 delivery. Reduction in relative NKILA transcript was confirmed by RT‐PCR (n = 3). (D) Relative EZH1 and EZH2 expression in non‐treated (NTC) cell lysates were measured by western blot. *P < 0.05, **P < 0.01, ***P < 0.001 by one‐way ANOVA

To determine whether pharmacological inhibition of EZH2 disrupts homeostatic levels of NKILA, cell lines were treated with either UNC1999, a nonspecific inhibitor of EZH1 and EZH2 or tazemetostat, an EZH2‐specific inhibitor. Subsequent fluctuations in NKILA transcript levels were measured by both quantitative RT‐qPCR using commercially available primers and semi‐quantitative RT‐PCR using alternative NKILA primers (data not shown). Inhibition of EZH2/1 and EZH2‐only in HMECs led to a reduction in NKILA transcript levels, with the most significant decrease observed upon nonspecific EZH2/1 inhibition. In contrast, EZH2/1 inhibition significantly increased NKILA transcript abundance in MCF‐7 cells, whereas inhibition of EZH2‐only demonstrated no appreciable effect. A two‐fold increase in NKILA expression was observed in MDA‐MB‐231 cells upon EZH2 inhibition, however, this effect was not reproduced under nonspecific EZH2/1 inhibition (Figure 2A).

Next, we confirmed the effects of EZH2/1 inhibition on EZH2 expression and activity by measuring EZH2 proteins levels and H3K27 methylation. As expected, we observed that nonspecific inhibition with UNC1999 reduces H3K27 methylation activity in all three cell lines. In contrast, EZH2 expression remained unchanged in HMECs, but showed a slight to moderate decrease in MDA‐MB‐231 and MCF‐7 cells, respectively. In MCF‐7 cells, decreased EZH2 expression correlates with reduced nuclear NF‐κB availability under EZH2/1 inhibition (Figure 2B). To investigate whether direct fluctuations in NKILA expression alter homeostatic levels of EZH2 and NF‐κB, cells were treated with miR‐103, a known negative regulator of NKILA. Partial silencing of NKILA significantly increased NF‐κB, EZH2 and H3K27 methylation in both breast cancer cell lines. In contrast, partial silencing of NKILA in HMECs reduced EZH2 levels and stimulated EZH2‐independent H3K27 methylation (Figure 2C).

As inhibition of EZH2/1 and EZH2‐only elicited diametric effects on NKILA expression in the two breast cancer cell lines, we explored the potential contribution of EZH1 to these cell‐specific events. While overall levels of EZH1 were higher in HMECs and MDA‐MB‐231 cells, EZH1 levels were more sensitive to EZH2 inhibition in the ER^+^ breast cancer cell line (Figure 2D).

### 3.3 Pharmacological inhibition of NF‐κB alters homeostatic levels of NKILA and EZH2 in a cell‐dependent manner

**Figure 3:**
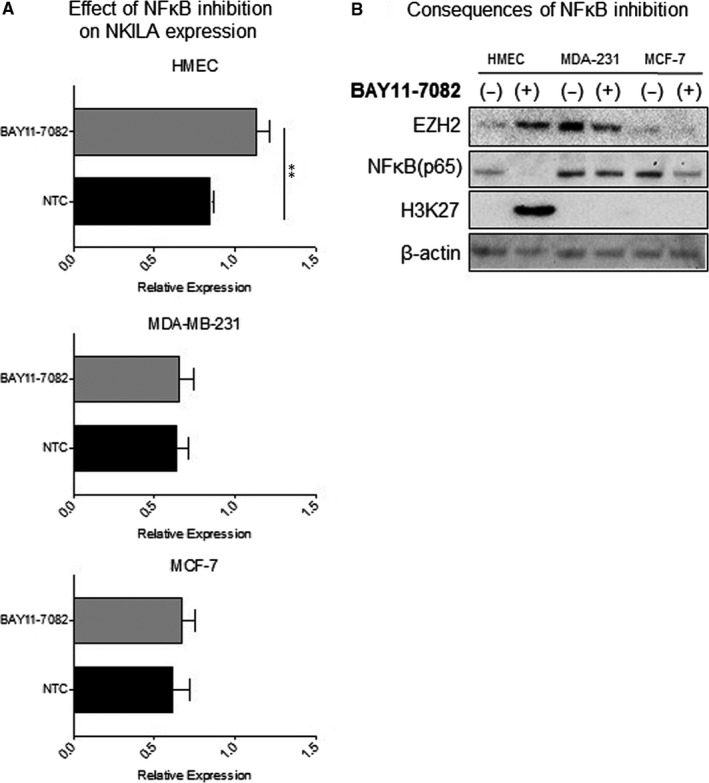
Consequences of NF‐κB restriction on NKILA and EZH2 expression and activity. (A) Measurement of relative NKILA transcript levels following NF‐κB inhibition by BAY11‐7082 (n = 3). (B) Western blots of nuclear cell lysates under NF‐κB restriction. **P < 0.01 by unpaired Student's *t *test

Next, we investigated the effects of nuclear NF‐κB depletion on NKILA homeostasis by treating cells with BAY11‐7082, an inhibitor of IκBα phosphorylation and NF‐κB nuclear translocation. Inhibition of NF‐κB significantly increased NKILA transcript levels in HMECs but had no effect on the two breast cancer cell lines (Figure 3A). We further examined the effects of NF‐κB inhibition on endogenous EZH2 expression and its methyltransferase activity. Silencing NF‐κB reduced EZH2 expression levels in both breast cancer cell lines, whereas the opposite effect was observed in HMECs. Inhibition of NF‐κB in the noncancerous cell line induced higher EZH2 expression and dramatically increased H3K27 methylation activity (Figure 3B).

### 3.4 Fluctuations in EZH2/NKILA/NF‐κB signaling lead to functional EMT remodelling

**Figure 4:**
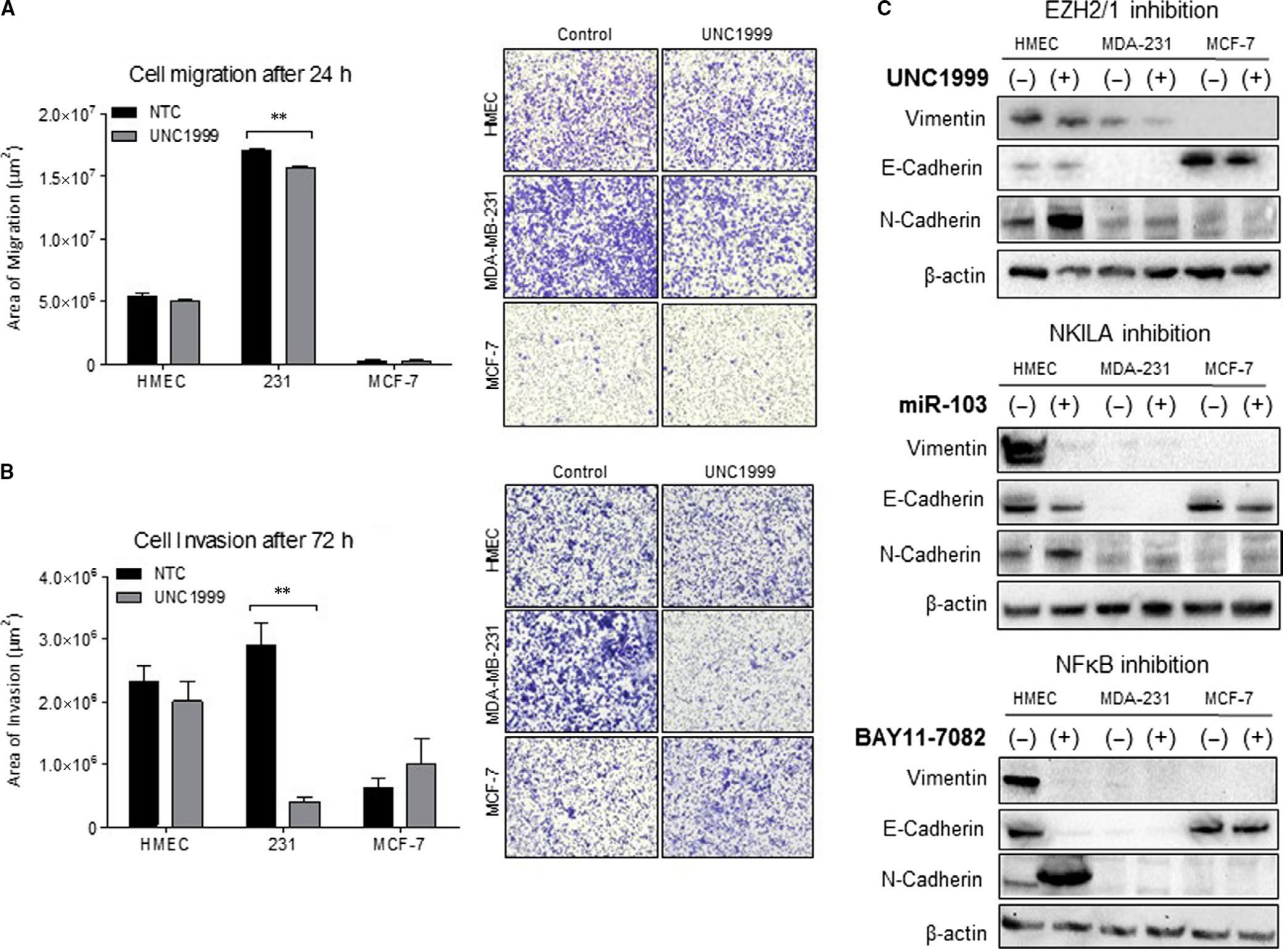
Functional effects of EZH2/1, NF‐κB and NKILA inhibition on cell motility and EMT. (A) Cell migration after 24 h and (B) Invasion through Matrigel after 72 h were assessed under EZH2/1 inhibition (n = 3). (C) Bright field images of cells following 48‐hour treatment with UNC1999 or tazemetostat. (D) Measurement of EMT markers in whole cell lysates following EZH2/1 inhibition, partial NKILA silencing and NF‐κB restriction. **P < 0.01 by unpaired Student's *t* test

We next investigated whether fluctuations in EZH2 expression functionally contribute to enhanced motility and invasiveness by measuring cell migration and invasion following EZH2/1 silencing. Inhibition of EZH2/1 significantly blunted MDA‐MB‐231 cell migration and invasion but had no observed effect on the motility of HMECs and MCF‐7 cells (Figure 4A and B).

We further explored the potential contribution of the EZH2/NF‐κB/NKILA signaling axis to the EMT process by probing EMT markers following respective silencing. Inhibition of EZH2/1 reduced the expression of Vimentin in MDA‐MB‐231 cells and E‐cadherin in MCF‐7 cells, while N‐cadherin expression was increased in HMECs. Partial NKILA silencing showed no major effect on EMT markers in either breast cancer cell line, however, miR‐103 delivery in HMECs dramatically attenuated the expression of Vimentin and E‐cadherin, while increasing N‐cadherin expression levels. Similarly, inhibition of NF‐κB augmented N‐cadherin expression and induced dramatic depletion of E‐cadherin and Vimentin in HMECs. However, NF‐κB inhibition had no observable effect on these markers in either breast cancer cell line (Figure 4C).

### 3.5 Pharmacological inhibition of NF‐κB, but not EZH2 alters cell proliferation in a growth factor‐dependent context

**Figure 5:**
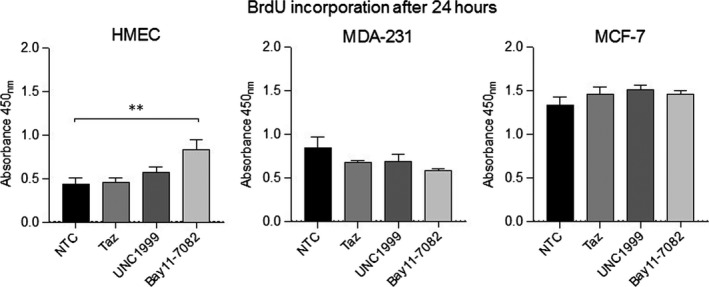
Cell proliferation following NF‐κB, EZH2‐only or EZH2/1 inhibition. BrdU incorporation was evaluated in cells treated with BAY11‐7082, tazemetostat, or UNC1999 in complete growth conditions (n = 4). **P < 0.01 by one‐way ANOVA

To determine whether fluctuations in NF‐κB and EZH2 influence cell proliferation, we assayed 5‐bromo‐2'‐deoxyuridine incorporation as a surrogate for measuring active cell division. Inhibition of NF‐κB significantly increased HMEC proliferation but had no appreciable effect on either breast cancer cell line. In contrast, EZH2‐only or EZH2/1 inhibition showed no significant effect on reducing cell proliferation in any of the three cell lines (Figure 5).

### 3.6 Inhibition of EZH2 contributes to cell fate response via nuclear NF‐κB depletion

**Figure 6:**
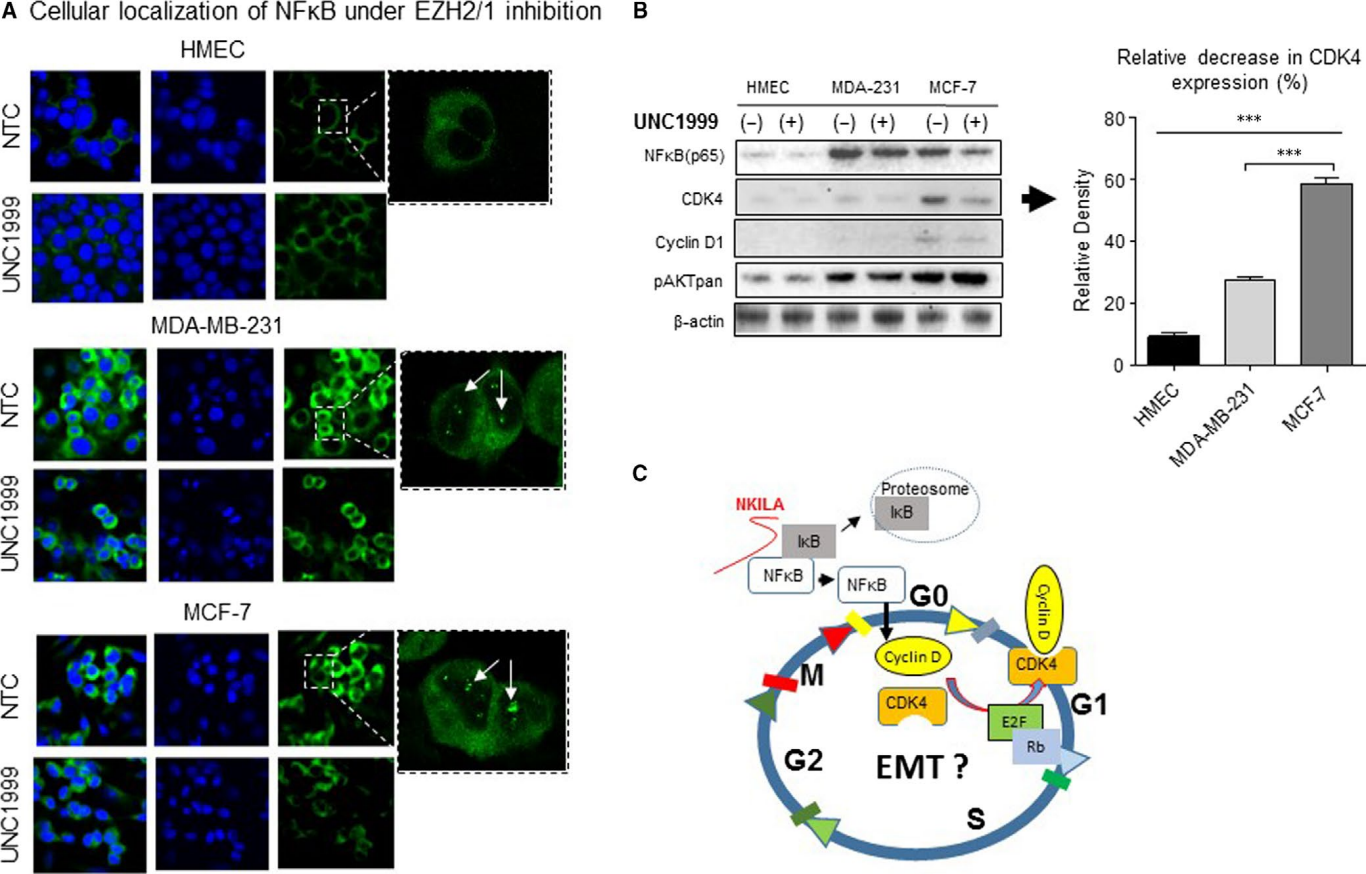
Effects of EZH2/1 inhibition on cell cycle regulation and cell fate. (A) Confocal images of immunofluorescence‐stained cells reveal NF‐κB localization under UNC1999 treatment. NF‐κB stained in green and nuclei stained with DAPI (blue). (B) Markers involved in cell cycle progression and cell fate response were measured following EZH2/1 inhibition. (C) Proposed interaction between NF‐κB/NKILA system components that drive cell cycle regulation ***P < 0.0001

We further evaluated the effect of EZH2 inhibition on NF‐κB‐mediated cell cycle progression and cell fate determination. Using immunofluorescence staining and confocal laser scanning microscopy, we confirmed that EZH2/1 inhibition reduced nuclear NF‐κB localization (Figure 6A). Next, we measured the expression of cell fate and cell cycle markers following EZH2 inhibition and subsequent NF‐κB restriction. As EZH2 is known to drive cell fate via ER‐dependent Wnt/β‐catenin signaling, we anticipated the most robust response to EZH2 inhibition in the ER^+^ MCF‐7 cells. Indeed, this appeared the case, as inhibition of EZH2/1 significantly decreased CDK4 expression and increased Akt phosphorylation, with the greatest effect observed in MCF‐7 cells (Figure 6B). Intriguingly, while all three cell lines experienced a reduction in CDK4 levels, no change to Cyclin D1 expression was observed. Based on these observations, we propose a signaling mechanism involving EZH2/NF‐κB‐mediated progression of cell cycle and downstream fate determination (Figure 6C).

### 3.7 Regulation of cell‐specific fate by NF‐κB is modulated by fluctuations in EZH2/NKILA and growth factor availability

**Figure 7:**
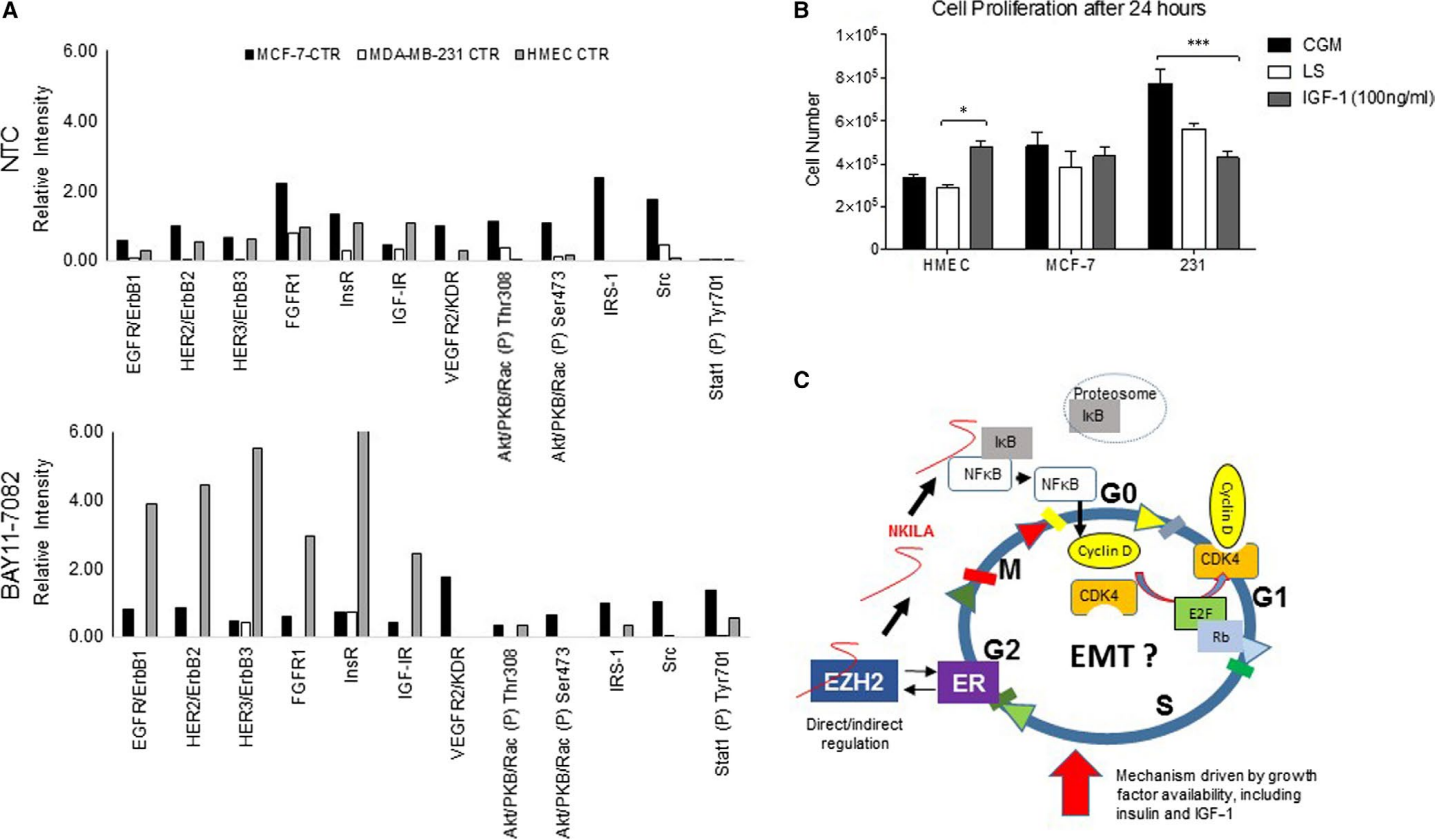
Identification of microenvironment cues regulating NF‐κB homeostasis. (A) Amplification of select RTKs under NF‐κB restriction (n = 2). (B) Cell proliferation under IGF‐1 delivery in low serum (LS) conditions compared to complete growth medium (CGM) positive control (n = 3). (C) Proposed schematic demonstrating functional regulation of the NKILA/NF‐κB system by EZH2 and extrinsic microenvironment cues, including growth factor availability. *P < 0.05, ***P < 0.001 by one‐way ANOVA

We further explored the involvement of growth factor signaling in NF‐κB‐driven cell proliferation by assessing RTK activity under NF‐κB restriction. As we previously observed an increase in NKILA transcript in HMECs following NF‐κB inhibition, we anticipated a robust RTK response in these cells compared to MCF‐7 and MDA‐MB‐231 cells. Indeed, the greatest fluctuations in RTK activity occurred in HMECs compared to either breast cancer cell line. We identified EGF, HER2, HER3, Fibroblast Growth Factor (FGF)‐1, Insulin and Insulin‐like Growth Factor (IGF)‐1 receptors as among the RTKs most highly up‐regulated following NF‐κB inhibition (Figure 7A). Treatment with IGF‐1 stimulated cell proliferation in HMECs, further confirming crosstalk between NF‐κB homeostasis and growth factor‐dependent cell cycle regulation (Figure 7B). Based on these results, we suggest a growth factor‐dependent mechanism for endogenous regulation of cell fate and migration by the EZH2/NF‐κB/NKILA signaling axis (Figure 7C).

## 4 DISCUSSION

The potential for interfering crosstalk among multiple regulators of NF‐κB signaling remains largely unexplored. Here, we investigated the interaction between EZH2 and the NF‐κB/NKILA signaling axis in a non‐tumourigenic breast epithelial cell line and in two breast cancer cell lines displaying differential hormone receptor expression.

Our evaluation of homeostatic levels of EZH2, NF‐κB and NKILA corresponds to expression levels established in previous studies of breast cancer cell lines and patient‐derived tumours. Constitutive activation of NF‐κB and EZH2 overexpression have been documented in highly aggressive breast carcinomas and are correlated with clinico‐pathological features.^28^, ^29^, ^30^, ^31^, ^32^ In contrast, breast cancer cell lines demonstrating high metastatic potential possess low levels of NKILA transcript, consistent with its suggested anti‐tumoural function.^25^ We anticipated and observed an inverse association between EZH2 expression and NKILA transcript levels, with respective high/low expression in breast cancer cells characterized by constitutively active NF‐κB and high EZH2 expression.

We confirmed the interdependence of EZH2, NF‐κB and NKILA by demonstrating significant and cell‐specific fluctuations in their expression following respective silencing of each signaling factor. As EZH1 is known to exert compensatory H3K27 methylation under EZH2 knockdown, we employed the use of an EZH2‐specific inhibitor and a nonspecific inhibitor to EZH2/1.^33^ EZH2/1 inhibition reduced nuclear NF‐κB expression, with the greatest reduction observed in ER^+^ breast cancer cells. The potentiated effect in these cells may be explained by ER_α_‐mediated mutual repression of NF‐κB, as both EZH2 and NF‐κB are known to interact directly with ER_α_.^34^

We observed opposing effects of EZH2 and EZH2/1 inhibition on NKILA expression among the three cell lines, suggesting direct and differential NKILA regulation by EZH2 and EZH1. EZH2‐specific inhibition significantly increased NKILA in MDA‐MB‐231 cells, suggesting a role for EZH2 as a negative regulator of NKILA transcription. In contrast, non‐specific inhibition of EZH2/1 in ER^+^ breast cancer cells may induce a more robust response over tazemetostat delivery alone, as EZH1 may negatively regulate NKILA under EZH2 inhibition. In contrast, EZH2 and EZH2/1 inhibition significantly attenuated NKILA transcript levels in HMECs, conferring a potential detriment to non‐transformed breast epithelial cells. It is important to note that NKILA transcript was measured with RT‐qPCR using commercially available NKILA primers. Considering its unique folding configuration, fluctuations in NKILA expression may vary depending on the specific NKILA primers used.

Reciprocal signaling between NKILA, EZH2 and NF‐κB was confirmed upon partial NKILA silencing, where we first observed evidence of EZH2‐independent induction of H3K27me activity in non‐transformed breast epithelial cells. As expected, partial NKILA silencing exerted a negative effect in both breast cancer cell lines by augmenting nuclear NF‐κB levels, EZH2 expression and H3K27 methylation.

Upon NF‐κB inhibition, non‐transformed mammary epithelial cells experienced a significant increase in NKILA transcript, EZH2 expression and methylation activity. These findings suggest differential regulation of NKILA by EZH2 and NF‐κB compared to the breast cancer cell lines. MDA‐MB‐231 cells were largely resistant to NF‐κB inhibition, however, its restriction induced significant fluctuations in EZH2 expression levels. This suggests a direct relationship between EZH2 and NF‐κB, in which the former is highly sensitive to fluctuations in the latter. This observation is supported by previous studies of ER^‒^ breast cancer cells, in which EZH2 was shown to interact non‐canonically with NF‐κB system components to potentiate NF‐κB transactivation.^21^

We investigated the influence of EZH2/1 restriction on cell motility and EMT to determine whether the EZH2/NF‐κB/NKILA signaling axis can be manipulated to enhance therapeutic intervention in breast cancer. Our finding that EZH2/1 restriction reduced the mobility and invasiveness of MDA‐MB‐231 cells is consistent with previous studies; however, this effect was not reproducible in MCF‐7 or HMECs, likely due to their comparatively low homeostatic EZH2 levels.^35^ Importantly, NKILA's known anti‐metastatic function results from its ability to inhibit cell migration, invasion, and EMT via various NF‐κB‐dependent pathways.^36^, ^37^ However, this did not proof in the MCF‐7 cells possibly due to their poor metastatic potential demonstrated by this particular cell line.

Perhaps most intriguing are our observations of the non‐transformed breast epithelial cell line. EZH2/1 inhibition induced the expression of the mesenchymal marker N‐cadherin, likely mediated by the loss of NKILA following EZH2/1 restriction. Indeed, this was confirmed following partial silencing of NKILA, whereby the gain in N‐cadherin expression was matched by loss of E‐cadherin and Vimentin. These expression patterns were mirrored with NF‐κB inhibition, further supporting alternative regulation of the EZH2/NF‐κB/NKILA signaling axis in non‐transformed cells compared to tumourigenic breast cells.

We further investigated the influence of NF‐κB and EZH2 inhibition on cell proliferation and showed a significant increase in HMEC proliferation following NF‐κB‐specific inhibition. However, EZH2‐only or EZH2/1 inhibition demonstrated no appreciable effect on proliferation of either breast cancer cell line. That may be explained in part by the absence of any known role exerted by NKILA on cell proliferation or differentiation. Indeed, no studies to date have reliably identified a proliferative function for NKILA. We observed a 50% decline in CDK4 expression in MCF‐7 cells, supporting knowledge that EZH2 is a known target of CDK4 and mediates MCF‐7 cell emergence. During early cellular senescence, EZH2 is down‐regulated and subsequently re‐expressed in dividing populations.^38^ As such, our observation that EZH2 restriction failed to influence cell proliferation may also be explained by insufficient treatment duration.

Host microenvironment factors, such as the bioavailability of EGF and IGF‐1 at indolent tumour sites, have been shown to modulate plasticity of certain breast tumours.^39^, ^40^ Thus, we interrogated RTK activity to further explain why NF‐κB inhibition stimulated significant HMEC proliferation. Of the three cell lines, HMECs experienced the greatest fluctuation in global RTK expression under NF‐κB inhibition. EGF, HER2, HER3, FGF, Insulin, and IGF‐1 receptors were among the RTKs most highly up‐regulated. Amplification of these receptors, individually and cooperatively, has been shown to promote tumour cell growth and is associated with breast carcinogenesis.^41^, ^42^, ^43^ Moreover, exogenous delivery of IGF‐1 significantly enhanced HMEC proliferation, suggesting the adoption of the IGF‐1 responsive phenotype observed by Castaño et al in triple negative tumours.

Cancer cell lineage variability observed in our study may be explained by at least four potential facets of the EZH2/NFkB/NKILA mechanism: (a) EZH2 and NFkB canonic versus non‐canonic activity which may differentially influence the action of NKILA from one cell type to another^14^, ^15^, ^16^, ^17^ (b) compensation of EZH2 enzymatic activity by the EZH1 isoform, (c) a chromatin‐independent function of EZH2 in immune‐homeostasis that may coincide with NFkB regulated cytokines^18^, ^36^, and (d) NKILA's regulatory effects on sensitizing T cells to activation‐induced cell death (AICD) by inhibiting NF‐κB.^18^ Mammary gland development and carcinogenesis are modulated by dynamic histone methylation landscapes, in addition to other well‐studied classical factors. The existence of direct and indirect interaction between EZH2, NF‐kB and NKILA may alter such mechanistic patterns in a cell lineage fashion. The reciprocity between NF‐κB/NKILA signaling changes the anti‐cancerous effects of EZH2 inhibition, and this too results in cell‐context disruption of homeostasis. Other groups have observed potentially deleterious effects of EZH2 inhibition on promoting transcriptional instability, EMT, and irreversible epigenetic reprogramming.^44^, ^45^, ^46^ By elucidating how EZH2 modulates the NF‐κB/NKILA mechanism, we identify cell‐intrinsic and systemic host elements that drive NF‐κB‐mediated imbalances, ultimately contributing to noncancerous cell transformation. Finally, this work supports future investigation into the EZH2/NF‐κB/NKILA signaling axis as a potential biomarker panel for monitoring therapeutic resistance under EZH2 inhibitor delivery, in addition to screening for personalized treatment regimens.

## CONFLICT OF INTEREST

There is no conflict of interest.

## AUTHOR'S CONTRIBUTION

CI coordinated all the experimental work and writing of the manuscript. SD and CI contributed to experimental design. SD, WKC, AO, DPB and CI performed experiments. SD, CI, and ILOB contributed to writing the manuscript. CMA and ILOB provided critical feedback for data interpretation.
